# Diarrhœa Successfully Treated by Sulphuric Acid

**Published:** 1852-12

**Authors:** 


*Diarrhœa Successfully Treated by Sulphuric Acid.*—Although Sulphuric Acid was recommended in passive diarrhœa, by the late Anthony Todd Thompson, in the Edition of his Dispensatory, published in 1837. I believe attention was only directed to it some twelve or fifteen months ago, by a writer in one of the Medical Periodicals; and according to received notions, no remedy would be less likely to check an attack of this disease, for the state of the tongue, the sour taste in the mouth, and the acid matters rejected from the stomach, would seem to contraindicate its use, and few practitioners venture to have recourse to any other remedies than the old ones of calomel and opium, or astringents and absorbents; which correct the acidity of the primæ viæ and improve the character of the secretions. I am induced to relate a few cases cured by this remedy, as I believe it will be found to be very efficacious in the treatment of this often troublesome disease.

*Case* 1*st.* James McCrae, aged 42, labourer, after exposure to wet, was attacked during the night of 22d June, with diarrhœa, succeeded after a time with severe griping pains in his bowels, accompanied with vomiting. Early in the morning, he procured from a neighboring apothecary shop, an ounce of castor oil, with 30 drops of laudanum, which he retained upwards of half an hour, when it was vomited up with a large quantity of very acid liquid, from which he felt much relieved for a few hours; when towards the middle of the day, all the symptoms having returned, with the same violence as in the morning, he came under my care, and I resolved to try the effect of Sulphuric Acid. I therefore, ordered a mixture containing ℥ss. of diluted Sulphuric Acid, with ℥viiss of water. ℥I to be taken every two hours. The first dose was vomited up within five minutes, but on taking the second which he did immediately on the rejection of the first, he had no return either of vomiting or griping pains, and the next dose checked the purging; he had no recurrence of it from that time, and the following day returned to his work as well as ever.

*Case 2nd.* Occurred a few days after the above, and was that of a lady who had just recovered from her accouchment, when she was attacked with nearly the same symptoms as the previous case; and was, when I first saw her, much exhausted from the enormous evacuations. I immediately ordered the same medicine, and two doses checked all the symptoms, although she took a third dose, as a matter of precaution, and now speaks to all her friends, of the acid mixture, as a specific in diarrhœa.

*Cases 3d and 4th.* Were in children—one two years old, and the other five; both were cured in two days, with the Sulphuric Acid, of course in much smaller doses, and sweetened at the time of administering it. I shall not detail many other cases, that have fallen under my care since these, but they have been of sufficient number to give me full opportunities of testing the value of sulphuric Acid in diarrhœa, and from the experience I have had in its use, I can confidently speak of its curative power in such cases.—*Canada Med. Jour.*

